# Sequence-Specific Detection of *Aristolochia* DNA – A Simple Test for Contamination of Herbal Products

**DOI:** 10.3389/fpls.2018.01828

**Published:** 2018-12-11

**Authors:** Tiziana Sgamma, Eva Masiero, Purvi Mali, Maslinda Mahat, Adrian Slater

**Affiliations:** ^1^Faculty of Health & Life Sciences, Biomolecular Technology Group, De Montfort University, Leicester, United Kingdom; ^2^Natural Product Testing Section, Toxic Compound Detection Unit, National Pharmaceutical Control Bureau, Jalan University, Selangor, Malaysia

**Keywords:** *Aristolochia*, *Stephania tetrandra*, DNA barcoding, herbal medicines, contamination, gBlock, quantitative real-time PCR

## Abstract

Herbal medicines are used globally for their health benefits as an alternative therapy method to modern medicines. The market for herbal products has increased rapidly over the last few decades, but this has in turn increased the opportunities for malpractices such as contamination or substitution of products with alternative plant species. In the 1990s, a series of severe renal disease cases were reported in Belgium associated with weight loss treatment, in which the active species *Stephania tetrandra* was found to be substituted with *Aristolochia fangchi*. *A. fangchi* contains toxic aristolochic acids, which have been linked to kidney failure, as well as cancers of the urinary tract. Because of these known toxicities, herbal medicines containing these compounds, or potentially contaminated by these plants, have been restricted or banned in some countries, but they are still available via the internet and in alternate formulations. In this study, a DNA based method based on quantitative real-time PCR (qPCR) was tested to detect and distinguish *Aristolochia* subg. *Siphisia* (Duch.) O.C.Schmidt species from a range of medicinal plants that could potentially be contaminated with *Aristolochia* material. Specific primers were designed to confirm that *Aristolochia* subg. *Siphisia* can be detected, even in small amounts, if it is present in the products, fulfilling the aim of offering a simple, cheaper and faster solution than the chemical methods. A synthetic gBlock template containing the primer sequences was used as a reference standard to calibrate the qPCR assay and to estimate the copy number of a target gene per sample. Generic primers covering the conserved 5.8S rRNA coding region were used as internal control to verify DNA quality and also as a reference gene for relative quantitation. To cope with potentially degraded DNA, all qPCR primer sets were designed to generate PCR products of under 100 bp allowing detection and quantification of *A. fangchi* gBlock even when mixed with *S. tetrandra* gBlock in different ratios. All proportions of *Aristolochia*, from 100 to 2%, were detected. Using standards, associating the copy number to each start quantity, the detection limit was calculated and set to about 50 copies.

## Introduction

Herbal medicines are often perceived as “good” and “safe” because they are “natural,” in contrast to “chemical” drugs. People tend to be more relaxed in using them and ask less questions of producers or practitioners. Unfortunately, it is a well-known fact that many plants are in fact toxic and dangerous (Efferth and Kaina, 2011). In some cases botanical misidentification of plants, deliberately or accidentally, can also play a role in herbal drugs toxic reactions.

In the early ‘90s, Han Fang Ji (*Stephania tetrandra*) was incorrectly substituted with Guang Fang Ji (*Aristolochia fangchi*) in diet pills probably because of their similar Chinese Pin Yin names (Vanherweghem et al., 1993; Nortier et al., 2000). *Aristolochia manshuriensis* (Guan Mu Tong) has also been reported to have been substituted for other Mu Tong herbal drugs which should have had contained *Akebia* and *Clematis* (Zhu, 2002; Yang et al., 2007). More recently, the substitution of *Solanum lyratum* by *Aristolochia mollissima* in Baiying preparations has been detected by DNA barcoding (Li et al., 2012).

Although *Aristolochia* species are used in Traditional Chinese Medicine (TCM) they are also known for containing nephrotoxic and carcinogenic aristolochic acids (AA) (Nortier et al., 2000; International Agency for Research on Cancer [IARC], 2002). AA have been classified as human carcinogenic class I by the World Health Organization International Agency for Research on Cancer in 2002 (International Agency for Research on Cancer [IARC], 2002). Because of this, herbal mixtures containing *Aristolochia* or plants that could be substituted with it because of similarities in their common names (i.e., *Stephania*, *Akebia*, *Asarum*, *Cocculus*, and *Sinomenium*), have been banned from the market (International Agency for Research on Cancer [IARC], 2002; Medsafe, 2003; Martena et al., 2007; Debelle et al., 2008; Abdullah et al., 2017). In spite of this, some of these species are still available in markets and via the internet and the risk of being contaminated with *Aristolochia* plants is still high (Schaneberg and Khan, 2004; Abdullah et al., 2017). In support of this are the hundreds of cases of renal failure linked to potential contamination by *Aristolochia* species that have been reported over the past two decades (Nortier et al., 2000; Debelle et al., 2008; Michl et al., 2013; Jadot et al., 2017).

There is still a tangible need for development of detection methods to avoid exposure to AA. A reasonable way to decrease this risk should be the systematic quality control of herbal preparations by using reproducible and accurate analytical methods. In the case of *Stephania* pills, herbal drugs are consumed in the form of ground roots. Although there are morphological differences between the roots of the genera described as Fang Ji, they also present many similarities which present the opportunity for mis-identification and substitution especially in powdered and macerated samples (Tankeu et al., 2016). For powdered samples, HPLC methods are used as they are considered to be more reliable (Joshi et al., 2008). Hyperspectral imaging studies that combine both chemical and physical properties have also been conducted in Fang Ji herbal medicines but the accuracy has a 10% limit in terms of prediction of adulteration (Tankeu et al., 2016). These methods all have limitations such as extensive sample preparation and being correlated to physiological influence, intraspecific differences and storage conditions. Quality control techniques that provide a rapid, inexpensive and accurate discrimination between the Fang Ji herbal medicines are still needed.

The practicality of using DNA barcoding in industrial quality assurance procedures has been recently discussed (Sgamma et al., 2017; Raclariu et al., 2018). Despite controversy around using DNA barcoding for herbal products authentication, DNA-based methods such as quantitative real-time PCR (qPCR), are a valuable addition to the toolkit of industrial quality assurance overcoming many of the limitations of standard DNA barcoding (Yang et al., 2018).

Different DNA-based methods for plants species identification and discrimination have attracted increased interest in recent year in many fields such as commercially processed food ingredients, spices, honey and herbal medicines. Species-specific qPCR assay has been proved to discriminate *Rhodiola rosea* from non-rosea Rhodiola species (Sgamma et al., 2017). Species-specific qPCR assays with Taq Man probes have been successfully used to discriminate several plants species in Corsican honey, while DNA metabarcoding and High Resolution melting analysis have been used to characterize the floral composition of honey in order to investigate honey bee foraging (Laube et al., 2010; Hawkins et al., 2015; Soares et al., 2018). High resolution melting (HRM) has been successfully used to differentiate seven selected *Zingiberaceae* plants (Osathanunkul et al., 2017). Duan et al. (2018) used barcoding coupled with HRM (Bar-HRM) to test the authenticity of *Rhizoma* species used in TCM as compared to their adulterants.

Focusing on the detection of *Aristolochia* species, a number of DNA-based methods, mostly targeting the *matK* and ITS2 regions, have been proved be promising in aiding in species-discrimination. Traditional DNA barcoding, targeting the chloroplast DNA loci *matK*, *rbcL* and *trnH-psbA* showed a different level of polymorphism between the loci with *matK* containing the most variation being able to discriminate genuine herbal medicines from their *Aristolochia* adulterants (Li et al., 2014). Yang et al. (2014) validated the ITS2 region as another DNA barcode region to discriminate *Aristolochia mollissima* from other plants used as herbal medicine including *Menispermi dauricum*, *Sophora tonkinensis*, *Stephania tetrandra*, and *Cocculus orbiculatus*. qPCR using TaqMan probes targeting the ITS2 region was also used to authenticate plant species from the Aristolochiaceae family and those from non-Aristolochiaceous substitutes and divide them in groups, but without quantifying the contamination (Wu et al., 2015). Loop-mediated isothermal amplification (LAMP) targeting the ITS2 region has also been proved to be effective in discriminating between Mu-tong, *Akebia caulis*, and its adulterant Guan-mu-tong, *Aristolochia manshuriensis* within 60 min in pure and mixed samples (Wu et al., 2016). More recently, Dechbumroong et al. (2018) developed a low cost and fast species-specific multiplex PCR assay to differentiate three *Aristolochia* species belonging to the subgenus *Aristolochia* (*Aristolochia pierrei*, *Aristolochia tagala*, and *Aristolochia pothieri*) present in Thailand known as “Krai-Krue.”

Here, DNA-based technology is proposed as a complementary approach to identify and quantify adulterant *Aristolochia* subg. *Siphisia* material in herbal formulations providing a reliable quality control for contamination of the plant material.

## Materials and Methods

### Plant Material and Total DNA Extraction

Fresh leaves or dry wood were provided by Dr Ben Gronier (De Montfort University, United Kingdom) and Prof Michael Heinrich (University College London, United Kingdom), respectively (Table 1). DNA was extracted from 100 mg of frozen material, previously ground to a fine powder in liquid nitrogen with mortar and pestle, using DNeasy Plant Mini Kit (Qiagen Inc., Germantown, MD, United States) following the manufacturers’ guidelines.

**Table 1:** Genetic material.

Species	Source	Kew DNA Bank ID	GenBank no. reference sequences
*Aristolochia kaempferi*	Dry wood- UCL		
*Aristolochia californica*	gDNA (Kew DNA Bank)	19176	
*Aristolochia baetica*	gDNA (Kew DNA Bank)	10534.1	
*Aristolochia clematitis*	gDNA (Kew DNA Bank)	13680	
*Stephania tetrandra*	gDNA (Kew DNA Bank)	25116	
*Stephania glandulifera*	Fresh leaves – DMU		
*Stephania rotunda*	Fresh leaves – DMU		
*Cocculus trilobus*	gDNA (Kew DNA Bank)	25115	
*Cocculus laurifolius*	gDNA (Kew DNA Bank)	39431	
*Sinomenium acutum*	gDNA (Kew DNA Bank)	25382	
*Asarum europaeum*	gDNA (Kew DNA Bank)	19154	
*Asarum arifolium*	gDNA (Kew DNA Bank)	198	
*Asarum fudsinoi*	gDNA (Kew DNA Bank)	21431	
*Saussurea alpine*	gDNA (Kew DNA Bank)	11885	
*Saussurea quercifolia*	gDNA (Kew DNA Bank)	44968	
*Diploclisia glaucescens*	gDNA (Kew DNA Bank)	1318	
*Menispermum dahuricum*	gDNA (Kew DNA Bank)	24519	
*Aristolochia fangchi*	gBlocks (IDT)		KP093067.1
*Stephania tetrandra*	gBlocks (IDT)		FJ609735.1
*Cocculus orbiculatus*	gBlocks (IDT)		AY864900.1
*Sinomenium acutum*	gBlocks (IDT)		AB571154.1


### DNA Samples

All genomic DNA (gDNA) samples were supplied pre-extracted from the Royal Botanic Gardens, Kew DNA Bank^1^ (Table 1).

### gBlock Fragments

**FIGURE 1:**
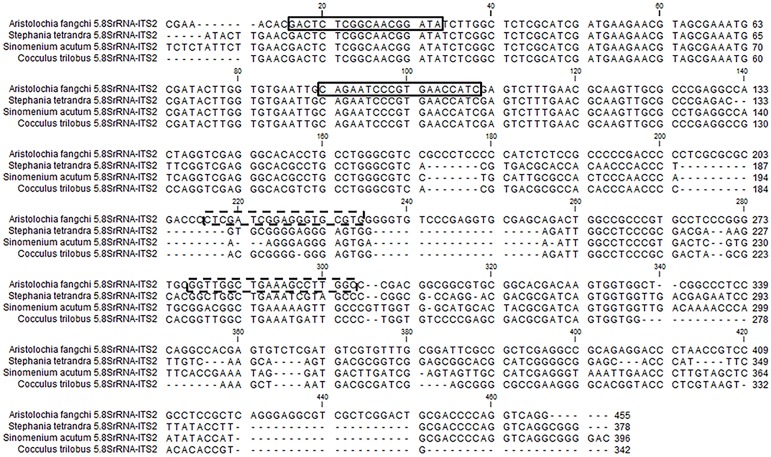
gBlock sequences alignment and primers location. The boxes show the generic 5.8S primers. The dotted boxes show the specific ITS2 primers located on the *Aristolochia fangchi* ITS gBlock sequence.

Four double-stranded, sequence-verified gene fragments, or gBlocks (Table 1), were ordered from Integrated DNA Technologies, BVBA (Leuven, Belgium). The gBlocks were designed to cover the 5.8S-ITS2 region within the nuclear ribosomal Internal Transcribed Spacer (nrITS) of the respective species (Figure 1). The GenBank accession numbers of the reference sequences are listed in Table 1. The gBlocks were resuspended in water at 10 ng/μl concentration. The copy number/μl in each gBlock was calculated converting the concentration from ng/μl to copy number/μl by using the formula provided by IDT guidelines ^2^ (Table 2). After optimisations, the S^-5^ dilution was used as working material.

**Table 2:** Quantification gBlock Fragments and DNA copy number.

gBlock	fmoles/ng	Calculated copy number/μl in Stock S	Calculated copy number/ μl in working dilution S^-5^
*Aristolochia fangchi*	3.56	2.14E + 10	2.14E + 05
*Stephania tetrandra*	4.28	2.58E + 10	2.58E + 05
*Cocculus orbiculatus*	4.73	2.85E + 10	2.85E + 05
*Sinomenium acutum*	4.09	2.46E + 10	2.46E + 05


### Phylogenetic Analyses

Phylogenetic analyses were conducted using the MEGA6.06 software package. The evolutionary history was inferred with the Maximum Likelihood method based on the Tamura 3-parameter model (Tamura, 1992).

### Primer Design

The NCBI database^3^ was accessed to obtain the nrITS sequences of *Aristolochia*, *Stephania*, *Cocculus*, and *Sinomenium*.

Based on all the nrITS sequences obtained, generic and *Aristolochia*-specific primers were designed (Table 3). The generic primers were designed to target the 5.8S conserved region while the specific primers were designed to the ITS2 region of selected problematic *Aristolochia* species (Figure 1). Primer specificity was determined using Basic Local Alignment Search Tool (BLAST) software^4^ and NCBI database (Supplementary Data Sheet S1).

**Table 3:** *Aristolochia*-specific and generic primers and annealing temperature (Ta) used in quantitative real-time PCR.

Primer name	Sequence	Ta	Expected size (bp)
*Aristolochia*-ITS2 F	5′- CTCGATCGGAGGGTGCGTG -3′	62°C	88
*Aristolochia*-ITS2 R	5′- GCCAAGGCTTTCAGCCAACC-3′		
Generic 5.8 F	5′- GACTCTCGGCAACGGATA-3′	60°C	93
Generic 5.8 R	5′- GATGGTTCACGGGATTCTG-3′		


### Standard PCR and Sequencing (nrITS)

PCR was performed using 1 × MyTaq Red Mix (Bioline), 0.2 μM of each forward (ITS1 TCCGTAGGTGAACCTGCGG) and reverse (ITS4 TCCTCCGCTTATTGATATGC) primers, and 1 μL of gDNA as template. Thermocycling conditions were optimized at 94°C for 2 min, followed by 40 cycles of 94°C for 15 s, 60°C for 30 s and 72°C for 30 s, with a final extension step of 72°C for 2 min. PCR products were run on 2% (w/v) agarose, 1 × TBE gels with 1 μL SYBR^®^ Safe DNA Gel Stain (Invitrogen, Paisley, United Kingdom) at 100 V for 30 min and analyzed in a Gel Doc^TM^ EZ Gel Documentation System (Bio-Rad, Oxford, United Kingdom). Products were submitted for sequence analysis to Macrogen^5^ to verify the authenticity of the starting material.

### Quantitative Real-Time PCR (qPCR)

Each qPCR reaction contained 1 × Sensifast SYBR green Hi-Rox mix (Bioline), 0.5 μl of gDNA or gBlock, 0.1 μM of each forward and reverse primer (Table 3), in a total volume of 10 μl made up with sterilized distilled water (SDW). qPCR was performed using three biological replicates with three technical replicates for each sample. After PCR amplification, all products were sequenced to confirm their identity. *Aristolochia* gBlock serial dilutions (from S^-3^ to S^-7^) were run to generate the standard curve (Supplementary Data Sheet S2). Working dilution gBlocks S^-5^, gDNAs and mixes of *Aristolochia* and *Stephania* gBlocks S^-5^ at different percentages and concentrations (Table 4) were used as templates. Water was run as a negative control for each test. A StepOnePlus^TM^ Real-Time PCR thermocycler machine (Applied Biosystem) was used. Thermocycling conditions were optimized at 95°C for 2 min, followed by 40 cycles of 95°C for 5 s and 30 s at the primer specific Ta (Table 3). The melting curve was obtained by melting the amplified template from 65 to 95°C increasing the temperature by 0.5°C per cycle. Analyses were conducted according to MIQE guidelines (Bustin et al., 2009). DNA levels were expressed as a relative proportion of the total DNA by using the generic primers as the “reference gene,” and compared to the control sample (*Aristolochia* working dilution gBlocks S^-5^) using the comparative (2^-ΔΔCt^) method (Livak and Schmittgen, 2001).

**Table 4:** Proportion of *Aristolochia* S^-5^ gBlock mixed with *Stephania* S^-5^ gBlock for contaminations tests and copy number in each dilution.

*Aristolochia* S^-5^ gBlock %	*Stephania* S^-5^ gBlock %	Copy number in neat (S^-5^) mixture		Copy number in 1:10 dilution		Copy number in 1:100 dilution		Copy number in 1:1000 dilution	
		*Aristolochia*	*Stephania*	*Aristolochia*	*Stephania*	*Aristolochia*	*Stephania*	*Aristolochia*	*Stephania*
100	0	2.14E + 05	0	2.14E + 04	0	2.14E + 03	0	2.14E + 02	0
90	10	1.97E + 05	2.19E + 04	1.97E + 04	2.19E + 03	1.97E + 03	2.19E + 02	1.97E + 02	2.19E + 01
50	50	1.18E + 05	1.18E + 05	1.18E + 04	1.18E + 04	1.18E + 03	1.18E + 03	1.18E + 02	1.18E + 02
10	90	2.53E + 04	2.28E + 05	2.53E + 03	2.28E + 04	2.53E + 02	2.28E + 03	2.53E + 01	2.28E + 02
2	98	5.14E + 03	2.52E + 05	5.14E + 02	2.52E + 04	5.14E + 01	2.52E + 03	5.14 E + 00	2.52E + 02


### Contamination Testing Using qPCR

A contamination test was performed where the *Aristolochia* gBlock S^-5^ working sample was mixed with the *Stephania* gBlock S^-5^ working sample at different proportions (Table 4). Each mix was also diluted 1:10, 1:100, and 1:1000. DNA copy numbers were also calculated (Table 4).

## Results

### Amplification of the gDNA Templates With ITS Generic Primers

**FIGURE 2:**
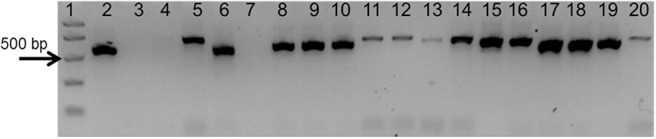
Agarose gel electrophoresis of PCRs using ITS1 and ITS4 generic primers. Gel lanes: (1) Easy Ladder I (Bioline); (2) Positive control; (3) Negative (no template) control; (4) *A. californica*; (5) *A. kaempferi*; (6) *A. baetica*; (7) *A. clematitis*; (8) *S. tetrandra*; (9) *S. glandulifera*; (10) *S. rotunda*; (11) *C. trilobus*; (12) *C. laurifolius*; (13) *S. acutum*; (14) *A. europaeum*; (15) *A. arifolium*; (16) *A. fudsinoi*; (17) *S. alpine*; (18) *D. glaucescens*; (19) *M. dahuricum*; (20) *S. quercifolia*.

To test the quality of the gDNA samples, a standard PCR using ITS1 and ITS4 primers was performed. The expected ITS fragment was detected in most of the samples (Figure 2). A very faint band was detected in *Aristolochia californica* (Figure 2, lane 4) and no bands were detected in *Aristolochia clematitis* sample (Figure 2, lane 7) (Figure 2). Identification of samples was confirmed by sequencing of the full ITS fragment.

### Phylogenetic Analysis

**FIGURE 3:**
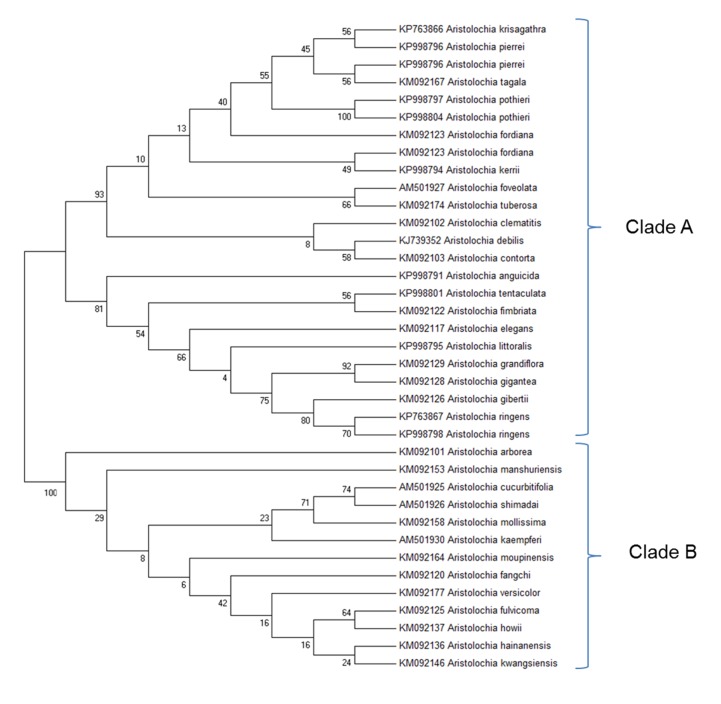
Molecular Phylogenetic analysis by Maximum Likelihood method. The evolutionary history was inferred by using the Maximum Likelihood method based on the Tamura 3-parameter model (Tamura, 1992). The bootstrap consensus tree inferred from 1000 replicates is taken to represent the evolutionary history of the taxa analyzed (Felsenstein, 1985). Branches corresponding to partitions reproduced in less than 50% bootstrap replicates are collapsed. The percentage of replicate trees in which the associated taxa clustered together in the bootstrap test (1000 replicates) are shown next to the branches (Felsenstein, 1985). Initial tree(s) for the heuristic search were obtained by applying the Neighbor-Joining method and BioNJ algorithms to a matrix of pairwise distances estimated using the Maximum Composite Likelihood (MCL) approach, and then selecting the topology with superior log likelihood value. The analysis involved 37 nucleotide sequences. All positions containing gaps and missing data were eliminated. There were a total of 127 positions in the final dataset. Evolutionary analyses were conducted in MEGA6 (Tamura et al., 2013). Accession numbers are given next to the species name.

Before designing *Aristolochia* primers the ITS2 regions of *Aristolochia* sequences present on NCBI GenBank database were aligned using the Clustal W MegAlign package of DNAStar (DNAStar Inc.). Evolutionary relationships of the genus members were inferred with the Maximum Likelihood method based on the Tamura 3-parameter model using the MEGA6.06 software package (Figure 3). The phylogenetic analysis showed two main clades. Species which have proved particularly problematic with regard to substitution and contamination, including *Aristolochia fangchi, A. manshuriensis* and *A. mollissima* were found to belong to Clade B. These two clades align with the two main *Aristolochia* subgenera (*Aristolochia* and *Siphisia*) supported by morphological and molecular studies (Ohi-Toma et al., 2006; Do et al., 2015; Wu et al., 2015), with the subgenus corresponding to Clade B correctly named as *Aristolochia* subg. *Siphisia* (Duch.) O.C.Schmidt (Ohi-Toma and Murata, 2016).

The clear separation between the two subgenera was apparent from examination of the multiple alignment of *Aristolochia* ITS2 sequences. The divergence between the sequences of the two subgenera was such that it proved difficult to design genus specific-primers that would amplify all members of the genus. This investigation therefore focused on the design of primers to detect the ITS2 sequences of some of the most problematic species, which belong to the subgenus *Siphisia*. These primers were designed to target regions in the ITS2 sequence that are very similar between all members of this subgenus, but differ from members of the subgenus *Aristolochia*. They can therefore be described as “*Aristolochia* subgenus *Siphisia*-specific” primers.

### Primers Specificity Testing Using Quantitative Real-Time PCR (qPCR)

Generic and *Aristolochia* subgenus *Siphisia*-specific qPCR primers were designed on the 5.8S and ITS2 region, respectively, within the nrITS sequence (Figure 1). Specificity of the developed qPCR reactions was evaluated in triplicate for each sample, including gDNA from various *Aristolochia* species and non-Aristolochiaceous genera including *Stephania*, *Cocculus, Sinomenium, Asarum, Saussurea, Diploclisia*, and *Menispermum.* Synthetic gBlocks designed to match *Aristolochia fangchi*, *Stephania tetrandra*, *Cocculus orbiculatus*, and *Sinomenium acutum* 5.8S and ITS2 regions were used as reference standards (Figure 1). The sensitivity of the triplex assay was determined using a serial dilution of *Aristolochia* gBlock DNA fragments representing the synthetic versions of the target genes at concentrations ranging from 0.1 to 1.00E-06 ng/μl per reaction. Linear regressions showed linear relationships (*r*^2^ = 0.999 for all runs) between the quantities of gBlock templates and the cycle threshold (Ct) values across the tested concentration range. The real-time PCR efficiency was 90.1 and 91.8, % for the generic internal control 5.8S and *Aristolochia* subgenus *Siphisia*-specific primers, respectively.

**FIGURE 4:**
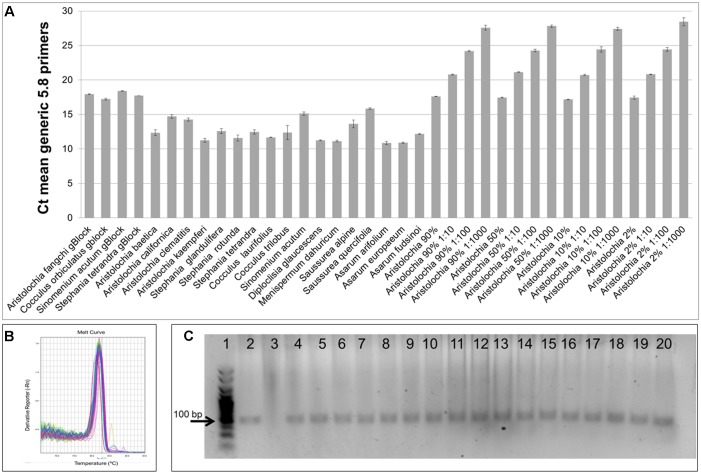
Generic internal control 5.8S quantitative real-time PCR. **(A)** Ct values. qPCR was performed using three biological replicates with three technical replicates for each sample. Error bars represent Standard deviation. **(B)** melting curve for all samples run with the generic 5.8S primers. **(C)** Agarose gel electrophoresis of PCRs using generic internal control 5.8S primers. Expected product size 93 bp. Gel lanes: (1) HyperLadder^TM^ 25 bp (Bioline); (2) Positive control; (3) Negative (no template) control; (4) *A. californica*; (5) *A. kaempferi*; (6) *A. baetica*; (7) *A. clematitis*; (8) *S. tetrandra*; (9) *S. glandulifera*; (10) *S. rotunda*; (11) *C. trilobus*; (12) *C. laurifolius*; (13) *S. acutum*; (14) *A. europaeum*; (15) *A. arifolium*; (16) *A. fudsinoi*; (17) *S. alpine*; (18) *D. glaucescens*; (19) *M. dahuricum*; (20) *S. quercifolia*.

As shown in Figure 4A, significant amplification signal was obtained in all samples when the generic primers were used. The Ct values in all gBlocks S^-5^ samples and the neat mixtures (*Aristolochia* plus *Stephania*) was between 17.2 and 18.4; the Ct value of the mixture dilutions was on average 20.8, 24.2, and 27.5 for the 1:10, 1:100, and 1:1000 dilutions showing an equivalent pattern when comparing DNA copy numbers. The Ct values of the genomic DNA samples were between 10.8 and 15.8. Primer specificity was assessed by melt curve analysis, with the results showing that just one peak was generated for all samples (Figure 4B). The size and uniformity of the product was confirmed visually by running the samples on agarose gel electrophoresis (Figure 4C). Interestingly, a product was visible in both *Aristolochia californica* and *Aristolochia clematitis* samples with a Ct value of 14.7 and 14.2, respectively. These two samples showed either a very faint or no band, respectively, when PCR was performed to amplify the full length nrITS fragment (Figure 2).

**FIGURE 5:**
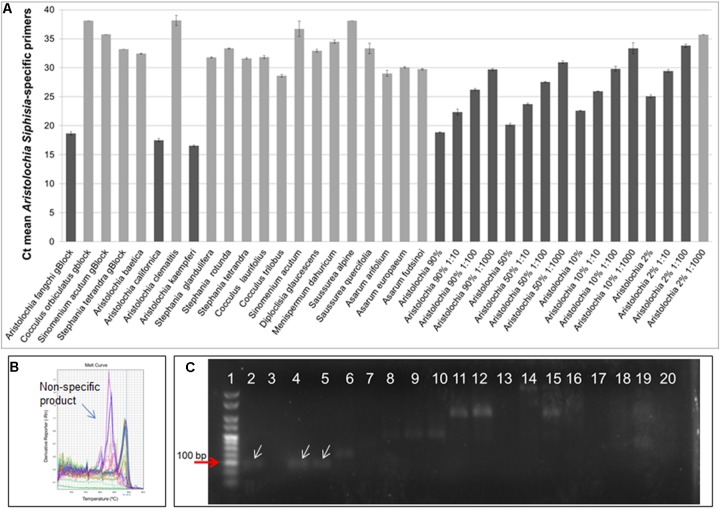
*Aristolochia* subgenus *Siphisia*-specific quantitative real-time **(A)** Ct values. The lighter bars indicate primer dimers or non-specific products as per melting curve data. qPCR was performed using three biological replicates with three technical replicates for each sample. Error bars represent Standard deviation. **(B)** Melting curve for all samples run with *Siphisia*-specific primers. **(C)** Agarose gel electrophoresis of PCRs using *Siphisia*-specific primers. The white arrows point to the products with the expected size (88 bp). Gel lanes: (1) HyperLadder^TM^ 25 bp (Bioline); (2) Positive control; (3) Negative (no template) control; (4) *A. californica*; (5) *A. kaempferi*; (6) *A. baetica*; (7) *A. clematitis*; (8) *S. tetrandra*; (9) *S. glandulifera*; (10) *S. rotunda*; (11) *C. trilobus*; (12) *C. laurifolius*; (13) *S. acutum*; (14) *A. europaeum*; (15) *A. arifolium*; (16) *A. fudsinoi*; (17) *S. alpine*; (18) *D. glaucescens*; (19) *M. dahuricum*; (20) *S. quercifolia*.

DNA copy numbers and Ct values obtained from qPCR using *Aristolochia* subgenus *Siphisia*-specific primers were used to compare specificity between target and non-target samples. The amplification plots for the *Siphisia*-specific primers showed a clear difference in Ct value (around 15 cycles) between the *Aristolochia* S^-5^ gBlock dilution and the S^-5^ dilution of non-target gBlocks (Figure 5A). The melting curve showed the presence of primer dimers and non-specific products with Ct values around and greater than 30 in gDNA non-target samples (Figure 5B). The presence of non-specific products in gDNA non-target samples was also confirmed visually running the samples on agarose gel electrophoresis (Figure 5C). The expected size product (88 bp) was visible in *Aristolochia* samples belonging to the subgenus *Siphisia* (*Aristolochia californica* and *Aristolochia kaempferi)* while the expected product was not visible in *Aristolochia* samples belonging to the subgenus *Aristolochia* (*Aristolochia baetica* and *Aristolochia clematitis*) or in the non-Aristolochaceous samples.

### Relative Quantitative Analysis of *Aristolochia* in Mixed Samples

To verify the feasibility of our method in the detection and quantitation of possible *Aristolochia* contamination in mixed samples, a series of gBlock admixtures containing different amounts of *Stephania tetrandra* (10, 50, 90, and 98% respectively) and model adulterant *Aristolochia fangchi* DNA were prepared (Table 4) starting from the gBlocks S^-5^ dilution. Each of these mixtures was then diluted 1:10, 1:100, and 1:1000 to check detection limits (Table 4).

**FIGURE 6:**
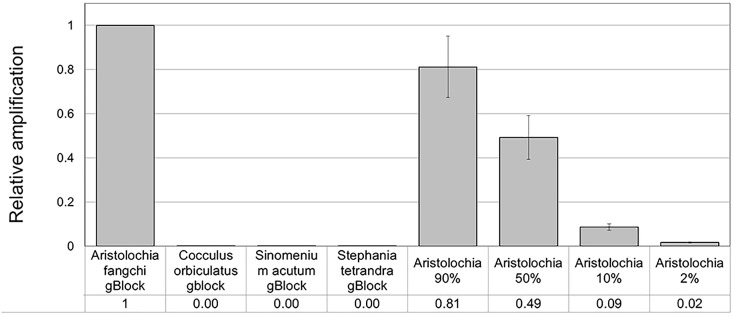
Detection and quantitation of *Aristolochia* subgenus *Siphisia* DNA using the *Siphisia*-specific primers. ΔCt values were calculated as the difference between the mean Ct value of the *Siphisia*-specific amplification and the mean Ct value of the internal control 5.8S amplification. *Aristolochia* S^-5^ gBlock was used as calibrator sample. qPCR was performed using three biological replicates with three technical replicates for each sample. Error bars represent Standard deviation.

**FIGURE 7:**
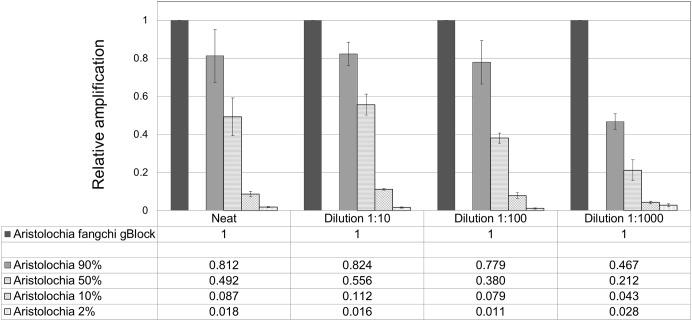
Quantitation of *Aristolochia* subgenus *Siphisia* DNA in admixtures. ΔCt values were calculated as the difference between the mean Ct value of the target *Siphisia*-specific amplification and the mean Ct value of the internal control 5.8S amplification. *Aristolochia* S^-5^ gBlock neat and dilutions 1:10, 1:100, and 1:1000 were used as calibrator samples for the corresponsive dilution mix. qPCR was performed using three biological replicates with three technical replicates for each sample. Error bars represent Standard deviation.

When comparing the relative proportions of *Aristolochia* DNA between the *Aristolochia* gBlock, the other species gBlocks and the mixtures representing the different contamination rates, consistent results were observed for all samples (Figure 6). The *Aristolochia* subgenus *Siphisia*-specific primers were able to detect *Aristolochia* DNA down to a 2% contamination level, while no amplification was detected in non-target gBlock samples. DNA copy numbers from the “100% contamination” sample (*Aristolochia* gBlock S^-5^) were used as the reference for relative DNA copy number calculation. Figure 7 shows that the serial dilutions showed the same pattern. Putting together this data and the samples melting curve results (Figure 5B) it is possible to set a safe detection limit of 2% for the 1:100 dilution which corresponds to a copy number of about 50 copies of *Aristolochia* *fangchi* nrITS DNA. In contrast, the 2% mixture in the 1:1000 dilution set gave a melting curve profile that indicated possible primer dimer formation. The detection limit is further supported by the detection of the correct amplicon in the 10% contamination mixture of the 1:1000 dilution set which would correspond to about 25 copies of *Aristolochia* nrITS DNA. Although the lowest dilution set of 1:1000 is at the limits of quantitative detection, it is still a useful qualitative indicator of the presence of *Aristolochia* at the lowest % mixtures, but not accurate enough to reliably quantify the amount of contamination.

### Testing gDNA

**FIGURE 8:**
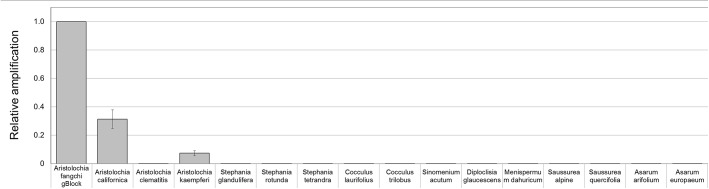
Detection and quantitation of *Aristolochia* subgenus *Siphisia* DNA sequences in genomic DNA samples. ΔCt values were calculated as the difference between the mean Ct value of the target *Aristolochia* *Siphisia*-specific amplification and the mean Ct value of the internal control 5.8S amplification. *Aristolochia* S^-5^ gBlock was used as calibrator sample. qPCR was performed using three biological replicates with three technical replicates for each sample. Error bars represent Standard deviation.

To prove that the detection and quantification test is valid with gDNA, a set of Aristolochiaceous and non-Aristolochiaceous genomic DNA samples were tested. All samples were amplified by the generic primers (Figure 4) indicating the absence of plant secondary product PCR inhibitors in the samples. Most of the samples also showed the presence of the full-length nrITS fragment (Figure 2), which was then sequenced to confirm the species. Although *Aristolochia californica* and *Aristolochia clematitis* did not show a clear band for the full-length nrITS fragment (Figure 2), they both acted as templates for the generic primers (Figure 4). None of the non-target species gDNA appeared to be amplified by the *Aristolochia* *Siphisia*-specific fragment (Figure 5), while the expected product was amplified in a range of target species in the *Aristolochia* subgenus *Siphisia* (Figure 5). The results were analyzed using the relative amplification method to determine the relative quantities of target species DNA compared to the amount of templates for the generic primers (Figure 8).

## Discussion

Aristolochic acid I and Aristolochic acid II have been identified as potent carcinogens and renal toxins (Arlt et al., 2002). All herbal formulations that contain any *Aristolochia* species have been classified as a Group 1 carcinogen by the International Agency for Research on Cancer (IARC) (International Agency for Research on Cancer [IARC], 2002; Grollman et al., 2007). Despite this classification it has been reported that products containing AA or suspected to contain AA are still in use and available on web sites (Gold and Slone, 2003; Nortier and Vanherweghem, 2007).

A reasonable way to detect the presence of *Aristolochia* contamination and decrease the risk associated with it, would be the systematic quality control of herbal preparations by using reproducible and accurate analytical methods. Currently, for industrial quality control, chemical and macro-morphology based analysis are conducted to identify the presence of *Aristolochia* species in herbal medicines (Kite et al., 2002; Lee et al., 2003; Sorenson and Sullivan, 2007; Joshi et al., 2008). These methods have limitations as they may be affected by many factors including growth conditions, environmental factors and post harvesting procedures (Zhang et al., 2012). DNA-based tests have emerged as a powerful, rapid, reliable, robust, and affordable identification system for authentication of medical plants and commercial herbal products that could be incorporated into industrial quality control processes (Sgamma et al., 2017).

Previous work has identified ITS2 as a suitable target to discriminate *Aristolochia* species and used 11 primer/probe combinations in TaqMan qPCR assay to identify herbal material from the Aristolochiaceae family and divide them in groups, but without quantifying the contaminant (Wu et al., 2015). Each combination of primers and probes detected different groups which contained some species from the Aristolochiceae family; for instance group A identified a large number of *Aristolochia* species but also many *Asarum* because they shared sequence similarity (Wu et al., 2015).

In this study, a simpler method was developed for the identification and quantification of the *Aristolochia* subgenus *Siphisia* in pure or mixed samples using DNA-based techniques designed to overcome the limitations of other identification methods. This was achieved by designing a reliable qPCR test to detect and quantify the presence of very small amounts of *Aristolochia* DNA using an internal control and an *Aristolochia* subgenus *Siphisia*-specific set of primers. qPCR is a simple, fast and sensitive test that could be suited to industrial quality control testing (Sgamma et al., 2017).

One of the limitations of working with banned herbal products is sourcing the samples and this study is not an exception. *Aristolochia fangchi* plant material or gDNA was unavailable. Therefore to overcome this issue, synthetic DNA, a gBlock, was designed based on the reference barcoding regions available in GenBank. The quality and quantity of many samples sourced through DNA banks is similarly a limitation. The amount of gDNA sample provided is usually in the order of few μls. The DNA concentration is also never very high, possibly due to the poor quality of the original plant material. Therefore, having enough material for optimizations and replicates is often an issue. The gDNA samples used in this study were therefore checked through barcoding the nrITS region. The sequence results gave an indication of DNA quality and also a prove of the authenticity of the samples. gBlocks were sourced also for the other plants species used in this study to overcome the problem related to the amount of DNA provided. Another reason for using gBlocks was to develop a quantification assay. Following the MIQE guidelines, when using qPCR for quantification rather than identification, it is necessary to generate a standard curve from known quantities of a target (Bustin et al., 2009).

Although quantification kits to be used as validated standards are commercially available, when working with non-human samples they became less reliable (Nielsen et al., 2006; Conte et al., 2018). Standard templates have been used from a range of sources, including cloned target sequences and PCR products, which require many steps that could potentially contaminate the laboratory and the standard itself. More recently, the use of synthetic gene fragments, such as gBlocks, as a standard has becoming an affordable, fast and reliable quantification strategy (Dhanasekaran et al., 2010; Conte et al., 2018).

In this study a gBlock has been used to create a standard curve to overcome the lack of available and reliable material, but also to prove that is possible to create a sensitive and reliable assay which can estimate the copy number of a target gene per sample and could potentially be used in an industrial setting. Reconstitution of the lyophilized gBlocks fragment provided over 2.14E+10 copies of the target. Dilution of the stock standard was done to create a sub-stock that was used to prepare the standard curve for the qPCR assay.

Generic primers were designed to target the conserved 5.8S rRNA coding region to amplify any template DNA. These can be used as an internal control to verify DNA quality and also as a reference gene for relative quantitation of the specific target DNA region. This primer pair was designed to generate a PCR product of under 100 bp which makes them suitable to be used in qPCR and ideal when working with potentially degraded DNA (Sgamma et al., 2017). This “mini-barcode” region proved to be useful for two of our samples. In fact, *Aristolochia californica* and *Aristolochia clematitis* gDNA samples did not present a clear amplicon for the nrITS fragments but then both of them presented templates for the generic 5.8 primers (Figure 4) indicating the presence of possible degraded, but still detectable DNA.

The ITS2 sequences for *Aristolochia* species available in GenBank demonstrated that the ITS2 region can be used to distinguish Aristolochiaceous species from their putative substitutes (non-Aristolochiaceae family) (Wu et al., 2015). In this study the *Aristolochia* species were separated into two clades using the ITS2 region. These two clades were recognized as corresponding to two subgenera previously reported, with Clade A corresponding to *Aristolochia* subgenus *Aristolochia* while Clade B corresponds to *Aristolochia* subgenus *Siphisia* (Ohi-Toma et al., 2006, Do et al., 2015; Ohi-Toma and Murata, 2016). Short “mini-barcode” regions within the ITS2 sequence were targeted for the design of *Siphisia*-specific primers because of the many reports of substitution of non-toxic plants with plants belonging to this subgenus, including *Aristolochia fangchi*, *A. manshuriensis*, *A. kaempferi*, *A. mollissima*, and *A. versicolor* (Debelle et al., 2008). Furthermore, it proved to be difficult to design *Aristolochia* subgenus *Aristolochia* specific primers because the ITS2 sequences within this group are more diverse than those in the *Siphisia* subgenus. Therefore, in this study we chose to work on the subspecies that included Fang Ji and Mu Tong to target the worst known cases of contamination.

Although significantly high DNA copy numbers were present in all of the gBlock and genomic DNA samples, only the target species showed the presence of the *Siphisia*-specific “mini-barcode” regions.

Optimization of qPCR with *Aristolochia* *Siphisia*-specific primers allowed detection and quantification of this genus in mixed samples containing also *Stephania tetrandra* in different ratios. When *Aristolochia* DNA was mixed with *Stephania* at different rates, it was possible to detect it in 2% ratio *Aristolochia* and 98% of *Stephania*. Using standards associating the copy number to each start quantity this corresponded to about 50 copies. All proportions of *Aristolochia*, from 100 to 2%, were detected. The melting curve data provided confirmation that there was only one amplification product. *Stephania*, *Sinomenium* and *Cocculus* gBlocks or gDNA samples were not amplified by qPCR when using the *Aristolochia* subgenus *Siphisia*-specific primers. Although the amplification curves indicated a small amount of apparent amplification of gDNA samples, it was considered to be negligible because the Ct values were higher than the blank and the melting curves confirmed non-specific product or primer dimer formation. Therefore, it was proved that it is possible to differentiate *Aristolochia* subg. *Siphisia* from the other genera using a DNA-based strategy in pure or mixed samples. The achievement of this study could be utilized by the manufacturers, importers and retailers of herbal products to conduct a preliminary safety test for all of their raw materials. After that stage, only samples that were positively identified to contain *Aristolochia* subg. *Siphisia* species will be further confirmed by chemical analysis. *Cocculus orbiculatus*, *Sinomenium acutum*, and *Stephania tetrandra* have been proven scientifically for their health benefits (Zhao et al., 2012; Bhagya and Chandrashekar, 2018). This study describes a rapid, sensitive qPCR test for the detection of *Aristolochia* species in the subgenus *Siphisia*. The assay is designed for use by industrial and regulatory quality control laboratories for screening of herbal drugs for contamination by those *Aristolochia* plants that have most frequently been implicated in the toxicity of adulterated medicines. This study represents the first phase of assay development in which the parameters have been optimized using pure components and gBlocks. The next phase will be to trial the assay using DNA extracted from herbal medicines to determine how robust the method is under conditions of PCR inhibitors and low quantities of poor quality fragmented DNA. The qPCR primers sets were in fact designed to generate PCR products of under 100 bp to cope with potentially degraded DNA. The introduction of reliable contamination tests into the supply chains of medicinal plants that are currently banned because of the risk of *Aristolochia* contamination will enhance the quality assurance of the safety of these herbs for consumption. This should help to restore consumer confidence and could eventually lead to the previous bans imposed on these harmless plant species being revoked by the regulatory authorities.

## Author Contributions

TS and AS conceived the project, designed and supervised the experimental strategy. TS, EM, and AS edited the paper. TS, PM, and MM performed the experiments. TS analyzed the data, and wrote most of the paper.

## Conflict of Interest Statement

The authors declare that the research was conducted in the absence of any commercial or financial relationships that could be construed as a potential conflict of interest.

## Footnotes

1 https://dnabank.science.kew.org/homepage.html

2 https://eu.idtdna.com/pages/education/decoded/article/tips-for-working-with- gblocks-gene-fragments

3 http://www.ncbi.nlm.nih.gov

4 http://blast.ncbi.nlm.nih.gov/Blast.cgi

5 http://www.macrogen.com
