# Supplementary material for: Sequence-Specific Detection of Aristolochia DNA – A Simple Test for Contamination of Herbal Products
Source: Front Plant Sci. 2018 Dec 11;9:1828. doi: 10.3389/fpls.2018.01828 (PMC6297175; doi:10.3389/fpls.2018.01828)
Supplement: Supplementary file 1 [file Data_Sheet_1.PDF]

- NCBI Home
- Sign in to NCBI
- Skip to Main Content
- Skip to Navigation
- About NCBI Accesskeys

NIH U.S. National Library of Medicine

NCBI National Center for Biotechnology Information

-
- My NCBI
- Sign in to NCBI
- Register
- Sign Out

Primer-BLAST» JOB ID:HhTBsOok54zAtvGz_NPVgYbIxLOr29-uqg
Primer-BLAST Results
[?]

Primer-BLAST was developed at NCBI to help users make primers that are specific to intended PCR target. It uses Primer3 to design PCR primers and then uses BLAST and global alignment algorithm to screen primers against user-selected database in order to avoid primer pairs (all combinations including forward-reverse primer pair, forward-forward as well as reverse-reverse pairs) that can cause non-specific amplifications. To cite Primer-BLAST or look for more details, please consult our publication:

Ye J, Coulouris G, Zaretskaya I, Cutcutache I, Rozen S, Madden T (2012). Primer-BLAST: A tool to design target-specific primers for polymerase chain reaction. BMC Bioinformatics. 13:134.

Use of Primer3 itself is subject to the terms and conditions stated on Primer3 web site.

-

Input PCR template
none
Specificity of primers
Target templates were found in selected database: Nucleotide collection (nt)
Other reports
Search Summary

Detailed primer reports

Primer pair 1

	Sequence (5'->3')	Length	Tm	GC%	Self complementarity	Self 3' complementarity
**Forward primer**	CTCGATCGGAGGGTGCGTG	19	62.76	68.42	6.00	0.00
**Reverse primer**	GCCAAGGCTTTCAGCCAACC	20	62.44	60.00	5.00	1.00

Products on intended target

Products on allowed transcript variants

Products on potentially unintended templates

Products on target templates

>KM092177.1 Aristolochia versicolor voucher YC0661MT01 internal transcribed spacer 2, complete sequence

```
product length = 89
Forward primer  1   CTCGATCGGAGGGTGCGTG  19
Template        44  ...................  62

Reverse primer  1    GCCAAGGCTTTCAGCCAACC  20
Template        132  ....................  113
```

>KM092166.1 Aristolochia sp. YC0800MT02 internal transcribed spacer 2, complete sequence

```
product length = 89
Forward primer  1   CTCGATCGGAGGGTGCGTG  19
Template        44  ...................  62

Reverse primer  1    GCCAAGGCTTTCAGCCAACC  20
Template        132  ....................  113
```

>KM092165.1 Aristolochia sp. YC0800MT01 internal transcribed spacer 2, complete sequence

```
product length = 89
Forward primer  1   CTCGATCGGAGGGTGCGTG  19
Template        44  ...................  62

Reverse primer  1    GCCAAGGCTTTCAGCCAACC  20
Template        132  ....................  113
```

>KM092164.1 Aristolochia moupinensis voucher YC0675MT01 internal transcribed spacer 2, complete sequence

```
product length = 89
Forward primer  1   CTCGATCGGAGGGTGCGTG  19
Template        45  ...................  63

Reverse primer  1    GCCAAGGCTTTCAGCCAACC  20
Template        133  ....................  114
```

>KM092152.1 Aristolochia kwangsiensis voucher YC0660MT03 internal transcribed spacer 2, complete sequence

```
product length = 89
Forward primer  1   CTCGATCGGAGGGTGCGTG  19
Template        44  ...................  62

Reverse primer  1    GCCAAGGCTTTCAGCCAACC  20
Template        132  ....................  113
```

>KM092151.1 Aristolochia kwangsiensis voucher YC0660MT08 internal transcribed spacer 2, complete sequence

```
product length = 89
Forward primer  1   CTCGATCGGAGGGTGCGTG  19
Template        44  ...................  62

Reverse primer  1    GCCAAGGCTTTCAGCCAACC  20
Template        132  ....................  113
```

>KM092150.1 Aristolochia kwangsiensis voucher YC0660MT07 internal transcribed spacer 2, complete sequence

```
product length = 89
Forward primer  1   CTCGATCGGAGGGTGCGTG  19
Template        44  ...................  62

Reverse primer  1    GCCAAGGCTTTCAGCCAACC  20
Template        132  ....................  113
```

>KM092148.1 Aristolochia kwangsiensis voucher YC0660MT05 internal transcribed spacer 2, complete sequence

```
product length = 89
Forward primer  1   CTCGATCGGAGGGTGCGTG  19
Template        44  ...................  62

Reverse primer  1    GCCAAGGCTTTCAGCCAACC  20
Template        132  ....................  113
```

>KM092147.1 Aristolochia kwangsiensis voucher YC0660MT04 internal transcribed spacer 2, complete sequence

```
product length = 89
Forward primer  1   CTCGATCGGAGGGTGCGTG  19
Template        44  ...................  62

Reverse primer  1    GCCAAGGCTTTCAGCCAACC  20
Template        132  ....................  113
```

>KM092146.1 Aristolochia kwangsiensis voucher YC0660MT01 internal transcribed spacer 2, complete sequence

```
product length = 89
Forward primer  1   CTCGATCGGAGGGTGCGTG  19
Template        44  ...................  62

Reverse primer  1    GCCAAGGCTTTCAGCCAACC  20
Template        132  ....................  113
```

>KM092145.1 Aristolochia kwangsiensis voucher YC0660MT02 internal transcribed spacer 2, complete sequence

```
product length = 89
Forward primer  1   CTCGATCGGAGGGTGCGTG  19
Template        44  ...................  62

Reverse primer  1    GCCAAGGCTTTCAGCCAACC  20
```

```
Template        132  ....................  113
```

>[KM092143.1](KM092143.1) Aristolochia kaempferi f. heterophylla voucher YC0678MT03 internal transcribed spacer 2, complete sequence

```
product length = 89
Forward primer  1   CTCGATCGGAGGGTGCGTG  19
Template        46  ...................  64

Reverse primer  1    GCCAAGGCTTTCAGCCAACC  20
Template        134  ....................  115
```

>KM092140.1 Aristolochia kaempferi f. heterophylla voucher YC0678MT01 internal transcribed spacer 2, complete sequence

```
product length = 90
Forward primer  1   CTCGATCGGAGGGTGCGTG  19
Template        47  ...................  65

Reverse primer  1    GCCAAGGCTTTCAGCCAACC  20
Template        136  ....................  117
```

>KM092136.1 Aristolochia hainanensis voucher YC0676MT05 internal transcribed spacer 2, complete sequence

```
product length = 89
Forward primer  1   CTCGATCGGAGGGTGCGTG  19
Template        44  ...................  62

Reverse primer  1    GCCAAGGCTTTCAGCCAACC  20
Template        132  ....................  113
```

>KM092135.1 Aristolochia hainanensis voucher YC0676MT04 internal transcribed spacer 2, complete sequence

```
product length = 89
Forward primer  1   CTCGATCGGAGGGTGCGTG  19
Template        44  ...................  62

Reverse primer  1    GCCAAGGCTTTCAGCCAACC  20
Template        132  ....................  113
```

>KM092134.1 Aristolochia hainanensis voucher YC0676MT03 internal transcribed spacer 2, complete sequence

```
product length = 89
Forward primer  1   CTCGATCGGAGGGTGCGTG  19
Template        44  ...................  62

Reverse primer  1    GCCAAGGCTTTCAGCCAACC  20
Template        132  ....................  113
```

>KM092133.1 Aristolochia hainanensis voucher YC0676MT02 internal transcribed spacer 2, complete sequence

```
product length = 89
Forward primer  1   CTCGATCGGAGGGTGCGTG  19
Template        44  ...................  62

Reverse primer  1    GCCAAGGCTTTCAGCCAACC  20
Template        132  ....................  113
```

>[KM092132.1](KM092132.1) Aristolochia hainanensis voucher YC0676MT01 internal transcribed spacer 2, complete sequence

```
product length = 89
Forward primer  1   CTCGATCGGAGGGTGCGTG  19
Template        44  ...................  62

Reverse primer  1    GCCAAGGCTTTCAGCCAACC  20
Template        132  ....................  113
```

>[KM092121.1](KM092121.1) Aristolochia fangchi voucher YC0663MT02 internal transcribed spacer 2, complete sequence

```
product length = 88
Forward primer  1   CTCGATCGGAGGGTGCGTG  19
Template        46  ...................  64

Reverse primer  1    GCCAAGGCTTTCAGCCAACC  20
Template        133  ....................  114
```

>[KM092120.1](KM092120.1) Aristolochia fangchi voucher YC0663MT01 internal transcribed spacer 2, complete sequence

```
product length = 88
Forward primer  1   CTCGATCGGAGGGTGCGTG  19
Template        46  ...................  64

Reverse primer  1    GCCAAGGCTTTCAGCCAACC  20
Template        133  ....................  114
```

>[KP093067.1](KP093067.1) Aristolochia fangchi isolate SCBGP444_1 internal transcribed spacer 1, partial sequence; 5.8S ribosomal RNA gene and internal transcribed spacer 2, complete sequence; and 28S ribosomal RNA gene, partial sequence

```
product length = 88
Forward primer  1    CTCGATCGGAGGGTGCGTG  19
Template        378  ...................  396

Reverse primer  1    GCCAAGGCTTTCAGCCAACC  20
Template        465  ....................  446
```

>[HG004866.1](HG004866.1) Aristolochia sp. 2 MR-2013 genomic DNA containing 18S rRNA gene, ITS1, 5.8S rRNA gene, ITS2 and 28S rRNA gene, specimen voucher HITBC:Liana Mengsong 58_5_4

```
product length = 89
Forward primer  1    CTCGATCGGAGGGTGCGTG  19
Template        572  ...................  590
```

```
Reverse primer  1    GCCAAGGCTTTCAGCCAACC  20
Template        660  ....................  641
```

>GQ434591.1 Aristolochia kwangsiensis voucher PS0878MT01 internal transcribed spacer 2, partial sequence

```
product length = 89
Forward primer  1   CTCGATCGGAGGGTGCGTG  19
Template        44  ...................  62

Reverse primer  1    GCCAAGGCTTTCAGCCAACC  20
Template        132  ....................  113
```

>KM092149.1 Aristolochia kwangsiensis voucher YC0660MT06 internal transcribed spacer 2, complete sequence

```
product length = 89
Forward primer  1   CTCGATCGGAGGGTGCGTG  19
Template        44  .................C.  62

Reverse primer  1    GCCAAGGCTTTCAGCCAACC  20
Template        132  ....................  113
```

>KM092142.1 Aristolochia kaempferi f. heterophylla voucher YC0678MT04 internal transcribed spacer 2, complete sequence

```
product length = 90
Forward primer  1   CTCGATCGGAGGGTGCGTG  19
Template        45  .C.................  63

Reverse primer  1    GCCAAGGCTTTCAGCCAACC  20
Template        134  ....................  115
```

>KM092131.1 Aristolochia griffithii voucher YC0674MT01 internal transcribed spacer 2, complete sequence

```
product length = 89
Forward primer  1   CTCGATCGGAGGGTGCGTG  19
Template        44  ...C...............  62

Reverse primer  1    GCCAAGGCTTTCAGCCAACC  20
Template        132  ....................  113
```

>KM092179.1 Aristolochia versicolor voucher YC0661MT03 internal transcribed spacer 2, complete sequence

```
product length = 89
Forward primer  1   CTCGATCGGAGGGTGCGTG  19
Template        44  ...................  62

Reverse primer  1    GCCAAGGCTTTCAGCCAACC  20
Template        132  ...T................  113
```

>KM092178.1 Aristolochia versicolor voucher YC0661MT02 internal transcribed spacer 2, complete sequence

```
product length = 89
Forward primer  1   CTCGATCGGAGGGTGCGTG  19
Template        44  ...................  62

Reverse primer  1    GCCAAGGCTTTCAGCCAACC  20
Template        132  ...T................  113
```

>[KM092144.1](KM092144.1) Aristolochia kaempferi voucher YC0666MT01 internal transcribed spacer 2, complete sequence

```
product length = 90
Forward primer  1   CTCGATCGGAGGGTGCGTG  19
Template        43  ...................  61

Reverse primer  1    GCCAAGGCTTTCAGCCAACC  20
Template        132  .......G............  113
```

>[KM092139.1](KM092139.1) Aristolochia howii voucher YC0664MT03 internal transcribed spacer 2, complete sequence

```
product length = 89
Forward primer  1   CTCGATCGGAGGGTGCGTG  19
Template        44  ...................  62

Reverse primer  1    GCCAAGGCTTTCAGCCAACC  20
Template        132  ......C.............  113
```

>[KM092138.1](KM092138.1) Aristolochia howii voucher YC0664MT02 internal transcribed spacer 2, complete sequence

```
product length = 89
Forward primer  1   CTCGATCGGAGGGTGCGTG  19
Template        44  ...................  62

Reverse primer  1    GCCAAGGCTTTCAGCCAACC  20
Template        132  ......C.............  113
```

>[KM092137.1](KM092137.1) Aristolochia howii voucher YC0664MT01 internal transcribed spacer 2, complete sequence

```
product length = 89
Forward primer  1   CTCGATCGGAGGGTGCGTG  19
Template        44  ...................  62

Reverse primer  1    GCCAAGGCTTTCAGCCAACC  20
Template        132  ......C.............  113
```

>[KM092125.1](KM092125.1) Aristolochia fulvicoma voucher YC0679MT01 internal transcribed spacer 2, complete sequence

```
product length = 89
Forward primer  1   CTCGATCGGAGGGTGCGTG  19
Template        44  ...................  62

Reverse primer  1    GCCAAGGCTTTCAGCCAACC  20
```

```
Template        132  ......C............  113
```

>[KJ766113.1] Aristolochia mollissima voucher YC0514MT01 internal transcribed spacer 2, partial sequence

```
product length = 90
Forward primer  1   CTCGATCGGAGGGTGCGTG  19
Template        44  ...................  62

Reverse primer  1    GCCAAGGCTTTCAGCCAACC  20
Template        133  .......G............  114
```

>[JQ255434.1] Aristolochia mollissima voucher M. Li 49 internal transcribed spacer 1, partial sequence; 5.8S ribosomal RNA gene, complete sequence; and internal transcribed spacer 2, partial sequence

```
product length = 90
Forward primer  1    CTCGATCGGAGGGTGCGTG  19
Template        537  ...................  555

Reverse primer  1    GCCAAGGCTTTCAGCCAACC  20
Template        626  .......G............  607
```

>[JQ255433.1] Aristolochia mollissima voucher M. Li 48 internal transcribed spacer 1, partial sequence; 5.8S ribosomal RNA gene, complete sequence; and internal transcribed spacer 2, partial sequence

```
product length = 90
Forward primer  1    CTCGATCGGAGGGTGCGTG  19
Template        537  ...................  555

Reverse primer  1    GCCAAGGCTTTCAGCCAACC  20
Template        626  .......G............  607
```

>[AM501930.1] Aristolochia kaempferi f. heterophylla ITS1 (partial), 5.8S rRNA gene and ITS2 (partial)

```
product length = 89
Forward primer  1    CTCGATCGGAGGGTGCGTG  19
Template        470  ...................  488

Reverse primer  1    GCCAAGGCTTTCAGCCAACC  20
Template        558  .......G............  539
```

>[AM501928.1] Aristolochia kaempferi ITS1 (partial), 5.8S rRNA gene and ITS2 (partial)

```
product length = 90
Forward primer  1    CTCGATCGGAGGGTGCGTG  19
Template        479  ...................  497

Reverse primer  1    GCCAAGGCTTTCAGCCAACC  20
Template        568  .......G............  549
```

>[AM501926.1] Aristolochia shimadai ITS1 (partial), 5.8S rRNA gene and ITS2 (partial)

```
product length = 91
Forward primer  1    CTCGATCGGAGGGTGCGTG  19
Template        479  ...................  497

Reverse primer  1    GCCAAGGCTTTCAGCCAACC  20
Template        569  .......G............  550
```

>[AM501925.1](AM501925.1) Aristolochia cucurbitifolia ITS1 (partial), 5.8S rRNA gene and ITS2 (partial)

```
product length = 91
Forward primer  1    CTCGATCGGAGGGTGCGTG  19
Template        480  ...................  498

Reverse primer  1    GCCAAGGCTTTCAGCCAACC  20
Template        570  .......G............  551
```

>[KM092101.1](KM092101.1) Aristolochia arborea voucher YC0665MT01 internal transcribed spacer 2, complete sequence

```
product length = 90
Forward primer  1   CTCGATCGGAGGGTGCGTG  19
Template        46  .......C...........  64

Reverse primer  1    GCCAAGGCTTTCAGCCAACC  20
Template        135  .G..................  116
```

>[KU853283.1](KU853283.1) Aristolochia manshuriensis isolate 930 internal transcribed spacer 1, partial sequence; 5.8S ribosomal RNA gene, complete sequence; and internal transcribed spacer 2, partial sequence

```
product length = 89
Forward primer  1    CTCGATCGGAGGGTGCGTG  19
Template        310  TC.................  328

Reverse primer  1    GCCAAGGCTTTCAGCCAACC  20
Template        398  .G.C................  379
```

>[KU853282.1](KU853282.1) Aristolochia manshuriensis isolate 9-51 internal transcribed spacer 1, partial sequence; 5.8S ribosomal RNA gene, complete sequence; and internal transcribed spacer 2, partial sequence

```
product length = 89
Forward primer  1    CTCGATCGGAGGGTGCGTG  19
Template        310  TC.................  328

Reverse primer  1    GCCAAGGCTTTCAGCCAACC  20
Template        398  .G.C................  379
```

>[KU853281.1](KU853281.1) Aristolochia manshuriensis isolate 9-34 internal transcribed spacer 1, partial sequence; 5.8S ribosomal RNA gene, complete sequence; and internal transcribed spacer 2, partial sequence

```
product length = 89
Forward primer  1    CTCGATCGGAGGGTGCGTG  19
Template        310  TC.................  328
```

```
Reverse primer  1    GCCAAGGCTTTCAGCCAACC  20
Template        398  .G.C................  379
```

>KU853280.1 Aristolochia manshuriensis isolate 9-23 internal transcribed spacer 1, partial sequence; 5.8S ribosomal RNA gene, complete sequence; and internal transcribed spacer 2, partial sequence

```
product length = 89
Forward primer  1    CTCGATCGGAGGGTGCGTG  19
Template        310  TC.................  328

Reverse primer  1    GCCAAGGCTTTCAGCCAACC  20
Template        398  .G.C................  379
```

>KR025503.1 Aristolochia manshuriensis voucher 14JL111 5.8S ribosomal RNA gene and internal transcribed spacer 2, partial sequence

```
product length = 89
Forward primer  1   CTCGATCGGAGGGTGCGTG  19
Template        68  TC.................  86

Reverse primer  1    GCCAAGGCTTTCAGCCAACC  20
Template        156  .G.C................  137
```

>KM092157.1 Aristolochia manshuriensis voucher YC0469MT05 internal transcribed spacer 2, complete sequence

```
product length = 89
Forward primer  1   CTCGATCGGAGGGTGCGTG  19
Template        43  TC.................  61

Reverse primer  1    GCCAAGGCTTTCAGCCAACC  20
Template        131  .G.C................  112
```

>KM092156.1 Aristolochia manshuriensis voucher YC0469MT04 internal transcribed spacer 2, complete sequence

```
product length = 89
Forward primer  1   CTCGATCGGAGGGTGCGTG  19
Template        43  TC.................  61

Reverse primer  1    GCCAAGGCTTTCAGCCAACC  20
Template        131  .G.C................  112
```

>KM092155.1 Aristolochia manshuriensis voucher YC0469MT03 internal transcribed spacer 2, complete sequence

```
product length = 89
Forward primer  1   CTCGATCGGAGGGTGCGTG  19
Template        43  TC.................  61

Reverse primer  1    GCCAAGGCTTTCAGCCAACC  20
Template        131  .G.C................  112
```

>KM092154.1 Aristolochia manshuriensis voucher YC0469MT02 internal transcribed spacer 2, complete sequence

```
product length = 89
Forward primer  1    CTCGATCGGAGGGTGCGTG  19
Template        43   TC.................  61

Reverse primer  1     GCCAAGGCTTTCAGCCAACC  20
Template        131   .G.C................  112
```

>KM092153.1 Aristolochia manshuriensis voucher YC0469MT01 internal transcribed spacer 2, complete sequence

```
product length = 89
Forward primer  1    CTCGATCGGAGGGTGCGTG  19
Template        43   TC.................  61

Reverse primer  1     GCCAAGGCTTTCAGCCAACC  20
Template        131   .G.C................  112
```

BLAST is a registered trademark of the National Library of Medicine
Support center Mailing list YouTube

- National Library Of Medicine
- National Institutes Of Health
- U.S. Department of Health & Human Services
- USA.gov

NCBI

National Center for Biotechnology Information, U.S. National Library of Medicine 8600 Rockville Pike, Bethesda MD, 20894 USA
Policies and Guidelines | Contact
