# Supplementary material for: Sequence-Specific Detection of Aristolochia DNA – A Simple Test for Contamination of Herbal Products
Source: Front Plant Sci. 2018 Dec 11;9:1828. doi: 10.3389/fpls.2018.01828 (PMC6297175; doi:10.3389/fpls.2018.01828)

Standard curve *Aristolochia* gBlock serial dilutions (from S^-3^ to S^-7^) Generic 5.8 Primers


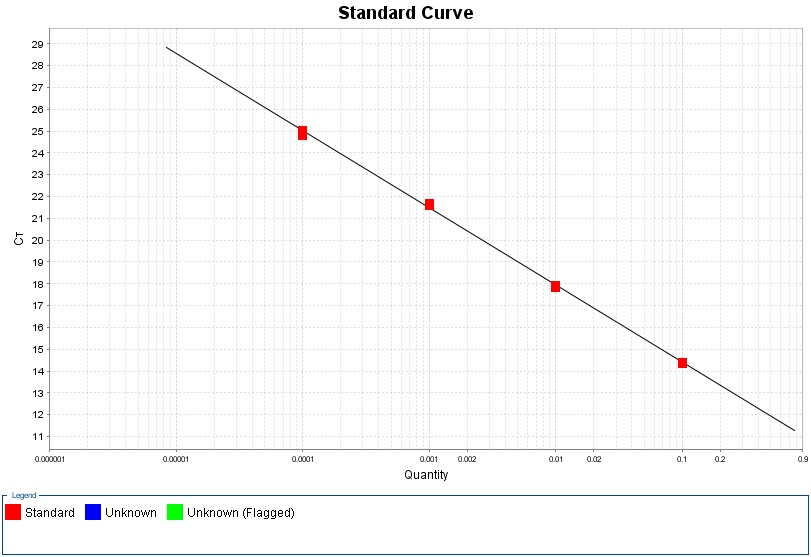


Standard curve *Aristolochia* gBlock serial dilutions (from S^-3^ to S^-7^) Aristolochia-ITS2 Primers


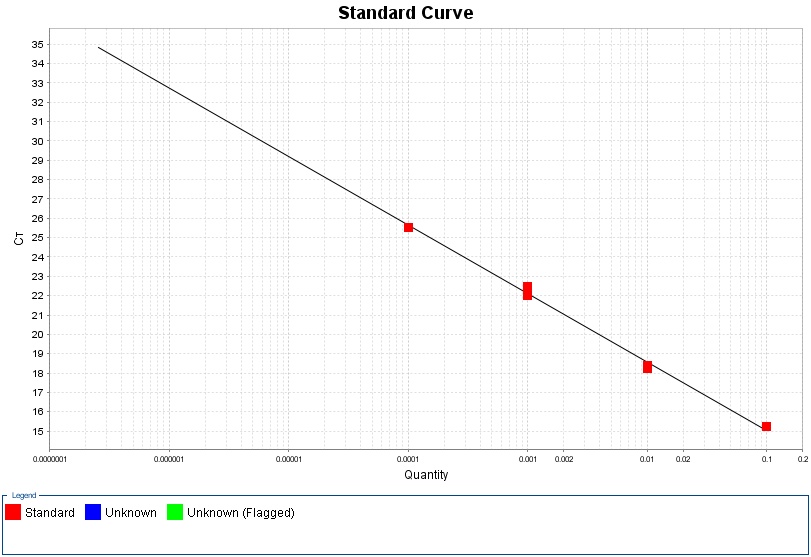

Supplement: Supplementary file 2 [file Data_Sheet_2.docx]
